# An Entangled Relationship between Bullying Perception and Psychosocial Dimensions in a Sample of Young Adolescents

**DOI:** 10.3390/children10111823

**Published:** 2023-11-17

**Authors:** Francesca Mastorci, Maria Francesca Lodovica Lazzeri, Paolo Piaggi, Cristina Doveri, Anselmo Casu, Gabriele Trivellini, Irene Marinaro, Caleb Devine, Cristina Vassalle, Alessandro Pingitore

**Affiliations:** 1 Clinical Physiology Institute, Consiglio Nazionale delle Ricerche, 56124 Pisa, Italy; francesca.mastorci@cnr.it (F.M.); m.francescalodovicalazzeri@gmail.com (M.F.L.L.); cristina.doveri@cnr.it (C.D.); gabriele.trivellini@cnr.it (G.T.); irene.marinaro@cnr.it (I.M.); caleb.devine.cd@gmail.com (C.D.); 2 Department of Information Engineering, University of Pisa, 56126 Pisa, Italy; paolo.piaggi@unipi.it; 3 Fondazione Toscana Gabriele Monasterio, 56124 Pisa, Italy; cristina.vassalle@ftgm.it

**Keywords:** bullying, victims, well-being, students, adolescence, HRQoL, school

## Abstract

Background: Bullying is a hostile behavior repeated over a time period, affecting children and adolescents in different social settings, mainly small and stable ones like school, with negative effects on mental and physical health. In this study, we aimed to provide the degree of impairment of different variables related to health and well-being in bullying conditions, with attention to sex differences. Methods: Data were obtained from 5390 adolescents (mean age 13.08 ± 1.89; male 2729), and health-related quality of life (HRQoL) was assessed using the KIDSCREEN-52 questionnaire. Results: In all students, mood and emotion, self-perception, and parental relationships are the dimensions more compromised in bullying conditions, while lifestyle habit is the variable less involved. Bullied girls show a significant impairment of all HRQoL variables both with respect to the socially accepted counterpart and to the male population. Conclusions: Our study highlights the strict association between bullying and emotional and social dimensions, suggesting that enhancing them preventively could facilitate earlier detection of problems, thereby reducing health risks.

## 1. Introduction

Bullying is a significant and prevalent problem among young people and presents in different contexts, including home, workplace, community settings, and school [1]. The term “bullying” refers to violent and intentional social behavior, not only physical but also verbal and psychological, direct (toward the target) or indirect (spreading rumors or images), with negative effects on psychological dimension, physical health, social relationships, and schoolwork. Usually, bullying takes place in a relatively small and constant setting, like classes, with the presence of the same people, and with almost daily events. For this reason, in recent years, there has been growing attention to the phenomenon of bullying in school settings, considering that approximately 1 in 5 school-aged youth have been victimized by this phenomenon. The American Medical Association and the National Academies of Sciences, Engineering, and Medicine identify bullying as a serious risk to children’s healthy development [2]. Although extensive research has well documented the consequences in the short-term on health and quality of life due to bullying victimization, considerably less is known about the reasons why the symptoms due to maltreatment persist even after the bullying has ceased, with effects on learning, behavior, and health [3]. It has also been seen that there is actually a kind of overlap between bullies and victims: those who behave as bullies during childhood may become victims in adolescence and vice versa [4]. In general, victims of bullying show problems regarding adaptation to stressful events, altered emotional responses such as depressed mood and anxiety, and psychosomatic diseases [5], not only in adolescence but also during their lifetime [6]. Lack of trust in social interactions would appear to be a predisposing factor to being victimized [7,8]. Bullying can cause a worse quality of life, as documented in previous studies; however, to our knowledge, there are no studies showing which psychosocial dimensions are most affected. The possibility of understanding which dimensions are most affected by bullying would make it possible to develop strategies to prevent and reduce the adverse effects of bullying in the short and medium terms [9,10].

Therefore, in light of this gap, the purpose of this study is to investigate how the perception of being a victim of bullying compared to those who feel socially accepted affects health-related quality of life and well-being. Accordingly, we paid attention to the different well-being-related dimensions and potential sex differences.

## 2. Materials and Methods

### 2.1. Study Population

Data were collected between 2022 and 2023 from the platform of the AVATAR project “A new purpose for promotion and eVAluation of healTh and well-being Among healthy teenageRs” developed by the Institute of Clinical Physiology of the NRC (National Research Council) [11]. This platform was designed to collect data on fundamental aspects of adolescent daily life, free of charge, without a commercial license, by the scholastic community (scholars, teachers, and parents) in order to improve adolescents’ overall self-esteem, resilience, and self-empowerment. Adolescent students were enrolled according to the following inclusion criteria: age 10–14 years, absence of neuropsychiatric or other diseases, informed consent signed, and filling of the entire questionnaires proposed.

Of the initial population of 5976, 586 students were excluded for the following reasons: diagnosed neuropsychiatric or other diseases (*n* = 20), absence of sign informed consent (*n* = 175), questionnaires not filled completely (*n* = 256), or internet connection problems (*n* = 135). Therefore, the final population consisted of 5390 adolescents. Participants were instructed on how to complete the questionnaires, and all tests were performed during school hours. In every school class, all the adolescents filled out the questionnaire, and whether they were not eligible due to exclusion criteria reasons were excluded from the study retrospectively. Participants were previously instructed on how to fill out the questionnaires and how to conduct the tests. One or two project members visited each school to provide the adolescents with verbal and written information about the data collection. All tests were conducted during participants’ computer lessons during school time. No incentive was provided to adolescents or parents. A research assistant was available to provide information and technical support to complete questionnaires.

### 2.2. Ethics

All parents or legal guardians gave informed consent and authorized researchers to use their data in accordance with Italian law. All procedures performed in the study were in accordance with the ethical standards of the institutional and/or national research committee and with the 1964 Helsinki Declaration and its later amendments or comparable ethical standards. The AVATAR project has been accepted by the Regional Pediatric Ethics Committee (Azienda Ospedaliero Universitaria Meyer) (16 February 2021, code 76).

### 2.3. Health-Related Quality of Life (HRQoL)

The Italian version of KIDSCREEN-52 was used to assess health-related quality of life (HRQoL), and data were acquired using the AVATAR platform [12,13]. A sociodemographic data record was used to collect information on gender and age.

The KIDSCREEN is a self-report questionnaire designed to address health-related quality of life, aimed to monitor and measure the personal experiences of children and adolescents about their perception of health status and well-being. The questionnaire, which describes physical, psychological, mental, social, and functional aspects of well-being, consists of 52 items grouped into 10 dimensions: physical well-being, psychological well-being, moods and emotions, self-perception, autonomy, parent relations, and home life, social support and peers, school environment, social acceptance (bullying), and financial resources [14]. Some sample items are “In general, how would you say your health is?” for the physical well-being dimension; “Have you felt satisfied with your life?” for moods and emotions; “Have you been happy with the way you are?” for self-perception. Cronbach’s alphas ranged from 0.77 to 0.89 for the dimensions of the 52-item version.

In further detail, physical well-being explores the level of the adolescent’s physical activity, energy, and fitness; psychological well-being examines the psychological well-being of the adolescent, including positive emotions and satisfaction with life; mood and emotions cover how much the adolescent experiences depressive moods and emotions and stressful feelings; self-perception includes whether the appearance of the body is viewed positively or negatively; autonomy looks at the opportunity given to an adolescent to create his/her social and leisure time; parent relations examine the relationship between the parents and the atmosphere in the adolescent’s home, with a focus on the quality of the interaction between the adolescent and parent or carer; social support and peers considers the nature of relations with friends and peers; school environment describes an adolescent’s perception of their cognitive capacity, learning, and concentration; social acceptance reflects the feeling of being rejected by peers in school; and financial resources describes the quality of the perceived financial resources [12,13,14].

With the exception of the mood and bullying dimensions, higher values of the variables express a better health-related quality of life. KIDSCREEN questionnaires were psychometrically tested using data obtained in a multicenter European study, which included a sample of 22,827 children recruited in 13 countries [14].

### 2.4. Psychological Well-Being Index

The Psychological Well-Being Index (PWBI) is composed of four components correlated with health-related well-being lifestyle habits (LH), emotional status (ES), social context (SC), and mental skills (MS) as perceived by adolescents [15]. The four dimensions were calculated and analyzed, and the different dimensions of KIDSCREEN-53 were collected via the questionnaire according to the structural model previously described by Mastorci and colleagues [16]. The procedure to obtain PWBI was described by Mastorci et al. [16].

### 2.5. Statistical Analysis

Statistical data analyses were executed using SPSS 27 software. Data are presented as mean  ±  SD or mean with a 95% confidence interval (CI). A *p*-value ≤ 0.05 was considered statistically significant. The Shapiro–Wilk test was used to assess the normality of data distribution for continuous variables before parametric analyses. The χ^2^ test was used for assessing the association between categorical variables. One-way between-groups multivariate analyses of variance were performed to evaluate differences for dimensions of health-related quality of life and PWBI between victims of bullying vs. nonvictims. In the case of overall significance for the Wilks’ Lambda statistic, follow-up analyses were conducted for each dimension of health-related quality of life and PWBI to assess inter-group differences using the Bonferroni adjustment of significance. Sensitivity analyses were also conducted, including sex as an additional independent variable in the multivariate model.

## 3. Results

### 3.1. Association between Perception of Bullying and HRQoL in Study Population and by Sex

In total, 5390 participants (50% girls, mean age 13.08 ± 1.89) were included in the analyses. Age was similar between males and females (male 13.06 ± 1.69 vs. female 13.43 ± 2.28, *p* = ns). The social acceptance population and bullied subjects did not differ numerically by sex (p calculated using the χ^2^ test). There was no sex difference between the two groups (χ^2^ = 0.20, *p* = 0.66). Table 1 shows the results of the multivariate analysis for the dimensions of HRQoL in the order of involvement between the two groups, namely socially accepted vs. bullied subjects; descriptive data by sex for HRQoL dimensions are presented in Table 2.

Bullying was globally associated with HRQoL dimensions (Wilks’ Lambda = 0.63, *p* < 0.001) and significantly associated with each dimension after adjustment for multiple tests (all adj. *p* < 0.05). Mood/emotion was the variable mostly affected by bullying perception (F = 349.9; adj *p* < 0.001), followed by self-perception (F = 166.7; adj *p* < 0.001), parent relationship (F = 164.4; adj *p* < 0.001), psychological well-being perception (F = 118.8; adj *p* < 0.001), and financial status (F = 118.3; adj *p* < 0.001). Moreover, the less altered but still affected variables were peer relations (F = 60.9; adj *p* < 0.001), physical well-being perception (F = 74.1; adj *p* < 0.001), school environment (F = 63; adj *p* < 0.001), and autonomy (F = 51.2; adj *p* < 0.001).

After adjustment for sex, the same results were maintained and expressed as the mean difference between bullying and social acceptance. Mood and emotion were always the variables most affected (adj. mean difference = −7.7, *p* < 0.001), followed by self-perception (adj. mean difference = −5.6 *p* < 0.001), parent relationship (adj. mean difference = −5.2 *p* < 0.001), financial status (adj. mean difference = −5.1 *p* < 0.001), psychological well-being perception (adj. mean difference = −4 *p* < 0.001), and peers’ relations (adj. mean difference = −3.8 *p* < 0.001). The variables less altered were autonomy (adj. mean difference = −2.9 *p* < 0.001), school environment (adj. mean difference = −2.7 *p* < 0.001), and physical well-being (adj. mean difference = −2.6 *p* < 0.001).

In social acceptance conditions, several variables significantly differed according to sex. Males perceived a higher physical (*p* < 0.001) and psychological well-being (*p* < 0.001), emotional status (*p* < 0.05), self-perception (*p* < 0.01), and autonomy (*p* < 0.001) compared to girls, who instead reported a better consciousness of financial resources (*p* < 0.001). There was also a sex difference regarding social context, in particular in a school environment where girls reported significantly higher levels than boys (*p* < 0.001). If we look at the bullying condition, boys’ victims of bullying continued to perceive better physical (*p* < 0.001) and psychological well-being (*p* < 0.001), mood and emotion responses (*p* < 0.05), and self-perception (*p* < 0.01) compared to girls, who once again showed a higher financial resources perception (*p* < 0.001).

### 3.2. Association between Perception of Bullying and Psychological Well-Being Score in Study Population and by Sex

The results of the association between social acceptance or bullying and PWBI are shown in Table 3, while in Table 4, they are divided by sex. The multivariate analysis indicates an overall effect of bullying on PWBI components (Wilks’ Lambda = 0.95, *p* < 0.001), with each component being significantly associated with bullying after adjustment for multiple tests (all adj. *p* < 0.005). In particular, according to the dimensions most involved, social context (family, school, and peers) was the component most affected by bullying perception (F = 207.30; adj. *p* < 0.001), followed by emotional state (F = 140.65; adj. *p* < 0.001) and lifestyle habits (F = 57.12; adj. *p* < 0.001). The following results were maintained after adjustment for sex: social context (adj. *p* < 0.001), emotional state (adj. *p* < 0.001), and lifestyle habits (adj. *p* < 0.001). When considering the association between bullying and PWBI as a function of sex, bullied girls reported significantly lower values of emotional state (*p* < 0.001) and lifestyle habits (*p* < 0.05) than boys.

## 4. Discussion

The aim of the study was to evaluate the relationship between the perception of being a victim of bullying and health-related quality of life in a sample of late adolescents. Consistent with the literature, bullying is generally associated with a reduction in well-being, without discriminating which dimension is predominantly affected. The contribution and innovation of our data are that the assessment is more detailed and almost offers a scale saying which dimension is most impaired in the bullied and which dimension is least involved in this dysfunctional behavior. The early point of this study showed that when we considered HRQOL or well-being index as a function of the two different groups and conditions—those who consider themselves socially accepted and those who perceive themselves to be bullied—the emotional component, self-perception, family relationships, and perceived well-being were the main dimensions altered by perception of bullying.

Furthermore, the association was much closer in females than in males. If the results were analyzed independently between the two groups, female adolescents had lower scores in the psychological area, in line with previous evidence obtained in the European sample, in which boys reported higher physical appearance, self-esteem, and mood dimensions [17,18,19,20].

**Figure 1:**
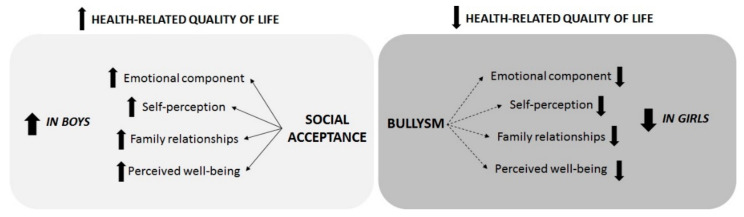
Descriptive results. Up arrow describes an increase/improvement; down arrow describes a decline.

Thus, the main findings of this study can be summarized in the following points: (i) the perception of being bullied mainly alters emotional and relational dimensions; (ii) the perception of bullying reduces the quality of life and well-being more in females than in males; and (iii) social acceptance enhances health-related dimensions more in males than in females (Figure 1).

These findings showed that in our sample, there were relevant associations between HRQoL and bullying, especially in psychological dimensions. However, previous results in this field are unclear, probably due to heterogeneous target populations, both clinical and healthy, by sex, and related to other different factors, such as cultural and country-specific ideology about the meaning of bullying [21,22,23].

This is an important aspect to consider, especially when the population is large. In fact, some cultural factors influence the idea and thus, the perception of bullying victimization so much that the meaning and the behavioral manifestations are different between countries [24]. Bullying is not a problem afflicting teenagers of the new generation from a physical, psychological, and social point of view, but rather a phenomenon that has always existed. Given the short- and long-term effects of the phenomenon, over the past decades, the community has been investigating the phenomenon from different perspectives with various and important projects [25].

A recent survey performed in Europe showed that 51% of students have experienced bullying in Lithuania, 50% of students in Estonia, 43% in Bulgaria, 31% in Greece, 25% in Latvia, and 15% in Italy, suggesting a growing dimension of bullying phenomenon in Europe communities today [25]. In a recent study conducted on 134,229 adolescents of 12 to 15 years of age, bullying victimization is considered a predictive factor of suicide attempts among adolescents globally, suggesting the urgent need to develop concrete and evidence-based interventions to address bullying and prevent psychological and health-negative outputs [26].

Another important point in the results, in line with the scientific literature in this area, is the fact that the whole female population, both bullied and socially accepted, have a lower HRQoL and well-being perception than their male counterpart, more pronounced in bullying victimization condition. On the other hand, our results showed a sex-related effect; that is, the perception of bullying in girls starting from a lower health-related quality of life values had a greater effect than in boys. These data are in line with previous studies, where adolescent girls exhibited lower mood and emotional reactivity and higher anxiety than boys [27,28]. Although there are no studies showing the role of bullying within depression symptomatology, it is considered a possible factor involved in its beginning and relapses across the lifespan [29,30]. One study conducted on 2680 adolescents demonstrated that among subjects with a history of bullying, depression was two times more common [31]. Contrary to the findings in our study, boys are more likely to be bullied; however, as they usually have higher HRQoL values, they may have less disruptive psychological effects than bullying perceived by girls who start from lower values. An additional stressor, such as being bullied, would certainly distort an already more compromised behavioral and emotional substratum. In particular, being bullied during elementary and middle school is considered an “early life stressor” that affects neurobiological development, resulting in an overactivity of the stress system with an increased vulnerability to neural abnormalities and depression in adulthood. In fact, the consequences of bullying on emotional dimensions and thus on mental health may persist over time. In this context, our data found that students who reported being bullied showed lower self-esteem and depressive-like behaviors, such as an impairment in social relations and a poorer well-being perception. These findings therefore suggest a compromised quality of life, intended as subjects’ perceptions of their position in life in the perspective of the culture and systems in which they live, concerning their objectives and expectations [32]. Thus, and our results confirm this, bullying victimization alters adolescents’ HRQoL and well-being perception, modifying physical health, psychological state, level of independence, social relations, and beliefs. Also, as documented in previous studies, a range of physical health problems (e.g., headaches and stomachaches) and somatic diseases (e.g., nausea and pain) are added to the effects documented in our study. Even the link between peer victimization and subject health symptoms, involving stress hyperreactivity, inflammation, and genetic biomarkers, was more pronounced in social victimization students compared to physical victimization [33]. Thus, the importance of the social context in the healthy development of adolescents emerges once again. In a previous study, we had, in fact, shown how the social context (family, school, and peers) modulates well-being and health status in its different dimensions: emotional, lifestyle, and cognitive [16]. Our results revealed that victims of bullying had impaired social relationships, both with family and friends. Accordingly, analyzing the personalized well-being index, defined as an integrated index of health-related variables, the social component was found to be the one most affected by the presence of bullying. At the same time, however, there is evidence showing that friendship and/or family support intercede between stress events and depressive symptoms; in other words, enhancing the relationship and social environments may benefit the health and well-being of vulnerable adolescents [34]. In fact, as our data showed, socially accepted subjects had strong relationships with family and friends without alterations in emotional sphere and mood. However, if social relationships are altered by bullying, it increases vulnerability to depression and mental health problems, creating a vicious circle.

As concerns the effects of bullying on lifestyle, data to our knowledge are scarce and conflicting, and much less attention has been given to the relationship between healthy habits and bullying. Our results, both in the form of individual variables and the integrated well-being index, showed that lifestyle habits are least affected by the experience of being bullied. Only few data obtained from the Health Behavior in School-Aged Children study examined the association between physical activity and sedentary behavior in terms of the risk of bullying victimization [35,36]. These results suggest that the lowest rates of bullying victimization are found among children and adolescents with a greater focus on physical education, probably because this promotes self-esteem and socialization and thus plays a protective role against becoming a victim of bullying [37].

From another perspective, there is evidence that subjects with a history of victimization during adolescence develop regulatory mechanisms to become more resilient in adulthood [38]. This compensatory response, postulated by Newman, would seem to be enacted only if, in the long-term, the same bullied subject is exposed to stress that mimics the social stress suffered in the past. According to this theory, such protective mechanisms mainly concern physiological activation during social stress; more specifically, it appears that these individuals have a blunted cardiovascular response, a typical pattern to reduce the susceptibility to develop noncommunicable diseases.

Considering the literature, some limitations of this study need to be considered [39,40,41,42]. First, no information related to categories of peer victimization, social/relational or physical, was acquired. Second, data regarding bullying victimization and HRQoL were collected on the basis of adolescents’ self-reports and subject to error and social bias. Furthermore, the study did not collect demographic information regarding living areas, whether rural or urban; in fact, this could be a factor influencing the perception of bullying. However, despite the enlarged use of “objective” health indicators, research in adolescents has been dominated by subjective (i.e., self-reported) health symptoms; certainly, also acquiring information from other informants could help us understand the results. Lastly, cultural factors that influence the meaning of bullying were not considered. In spite of these limitations, a major strength of our study was the large school samples representing very different geographic and cultural Italian settings.

## 5. Conclusions

The present study showed that bullying victimization reduced HRQoL mainly via the mediators of emotional and social dimensions. In addition, the results indicated that bullying was associated with an impairment of all health-related variables, more in girls than in boys. Furthermore, social relationships, encouraging communication among parents, children, and school, could facilitate earlier detection of problems, reducing subjective health risks. The research in this field shows antibullying interventions are effectively significant, with scientifically evaluated school-based programs and strategies documenting a reduction in bullying victimization of 20–30% [43].

Usually, whole-school programs are complex and multilevel, from students to the whole school), including a range of methodologies and variables, where the different components associated with bullying are considered in an integrated way and not separately [44,45].

Our findings, indicating which dimensions are most affected by the perception of bullying, provide an opportunity to prevent bullying in adolescence by potentiating specific behavioral coping strategies, such as self-esteem, social relationships, or physical education, in order to reduce health problems during one’s lifespan.

## Figures and Tables

**Table 1:** Associations between bullying perception and dimensions of HRQoL assessed via multivariate analysis of variance.

Dimensions	Mean Difference B-SA	95% Confidence Interval	F	*p*-Value
Bullying	−21.8	−22.4 to −21.1	3807.4	<0.001
Mood/Emotion	−7.4	−8.1 to −6.6	349.9	<0.001
Self-perception	−5.8	−6.6 to −4.9	166.7	<0.001
Parent relationship	−5.4	−6.3 to −4.6	164.4	<0.001
Psychological well-being	−4.4	−5.2 to −3.6	118.8	<0.001
Financial resources	−4.4	−5.2 to −3.6	118.3	<0.001
Peers	−3.3	−4.2 to −2.5	60.9	<0.001
Physical well-being	−3.2	−3.9 to −2.5	74.1	<0.001
School environment	−2.9	−3.7 to −2.2	63.0	<0.001
Autonomy	−2.8	−3.6 to −2.1	51.2	<0.001

Data given as mean with 95% confidence interval. Data on the KIDSCREEN-52 dimension are calculated according to KIDSCREEN group [12,13]. The order of the dimensions reflects how much more impactful the perception of bullying is, i.e., results on the rows are sorted in descending order for the mean difference between groups. B: Bullying perception; SA: Social Acceptance. *p*-values were adjusted based on Bonferroni formula.

**Table 2:** Health-related quality of life dimensions by social acceptance and bullying students and stratified by sex.

Dimensions	Social Acceptance		*p*-Value	Bullysm		*p*-Value	*p*-Value (B)	*p*-Value (G)
	Boys (*n* = 2465)	Girls (*n* = 2394)		Boys (*n* = 264)	Girls (*n* = 267)			
Physical wellbeing	49.6 ± 9.0	46.8 ± 8.5	<0.001	47.1 ± 9.3	43.3 ± 8.1	<0.001	<0.001	<0.001
Psychological wellbeing	49.2 ± 9.1	47.6 ± 9.9	<0.001	45 ± 10.5	42.7 ± 10.5	0.01	<0.001	<0.001
Mood/Emotion	48.3 ± 9.0	46.1 ± 9.9	<0.001	40.6 ± 10	38.6 ± 10.3	<0.001	<0.001	<0.001
Self-perception	52.6 ± 10.1	49.4 ± 11.4	<0.001	47.1 ± 9.8	43.6 ± 11.2	<0.001	<0.001	<0.001
Autonomy	46.8 ± 9.4	44.4 ± 9.5	<0.001	43.9 ± 9.7	41.8 ± 10.8	0.02	<0.001	<0.001
Parent relationship	50.7 ± 9.6	49.3 ± 10.7	<0.001	45.5 ± 9.6	43.0 ± 11.4	0.01	<0.001	<0.001
Financial resources	49.6 ± 9.7	50.9 ± 9.7	<0.001	44.5 ± 93	47.7 ± 10.1	<0.001	<0.001	<0.001
Peers	49.9 ± 10.7	49.1 ± 10.0	0.01	46 ± 10.5	46.1 ± 11.4	n.s.	<0.001	<0.001
School environment	48.3 ± 8.9	49.7 ± 8.9	<0.001	45.5 ± 8.7	45.8 ± 9.5	n.s.	<0.001	<0.001

Data given as mean ± SD. Data on the KIDSCREEN-52 dimension are calculated as the mean T-scores according to KIDSCREEN group [12,13]. Comparison B: Social Acceptance vs. Bullyism in boys; G: Social Acceptance vs. Bullyism in girls; n.s.: not significant (*p* > 0.05).

**Table 3:** Associations between bullying perception and components of the Psychological Well-Being Index assessed via multivariate analysis of variance.

Components	Mean Difference B-SA	95% Confidence Interval	F	*p*-Value
Social Context	−3.1	−3.6 to −2.7	207.30	<0.001
Emotional Status	−2.5	−2.9 to −2.1	140.65	<0.001
Lifestyle Habits	−1.1	−1.4 to −0.8	57.12	<0.001
Mental Skills	0.0	0.0 to 0.0	0.35	0.557

Data given as mean with 95% confidence interval. The order of the components reflects how much more impactful the perception of bullying is, i.e., results on the rows are sorted in descending order for the mean difference between groups. B: Bullying perception; SA: Social Acceptance. *p*-values were adjusted based on Bonferroni formula.

**Table 4:** Psychological Well-Being Index by social acceptance and bullying students and divided by sex.

PWBI Components	Social Acceptance		*p*-Value	Bullysm		*p*-Value
	Boys (*n* = 2465)	Girls (*n* = 2394)		Boys (*n* = 264)	Girls (*n* = 267)	
Social Context	18.3 ± 4.8	18.1 ± 4.5	n.s.	15.2 ± 4.0	14.9 ± 3.7	n.s.
Emotional Status	16.1 ± 4.5	15.2 ± 4.3	<0.001	13.5 ± 3.8	12.9 ± 3.9	n.s.
Lifestyle Habits	12.5 ± 3.1	12.1 ± 2.9	<0.001	11.4 ± 2.8	11.1 ± 2.5	n.s.
Mental Skills	1.2 ± 0.4	1.2 ± 0.4	n.s.	1.2 ± 0.4	1.2 ± 0.4	n.s.

Data given as mean ± SD. Data on the PWBI components are calculated as described in [16]. n.s.: not significant (*p* > 0.05).

## Data Availability

Available only upon request to the corresponding author.
